# Transgenic *Nicotiana benthamiana* plants expressing a hairpin RNAi construct of a nematode *Rs-cps* gene exhibit enhanced resistance to *Radopholus similis*

**DOI:** 10.1038/s41598-017-13024-9

**Published:** 2017-10-13

**Authors:** Yu Li, Ke Wang, Qisen Lu, Juan Du, Zhenyue Wang, Desen Wang, Bingjian Sun, Honglian Li

**Affiliations:** 1 Department of Plant Pathology, Henan Agricultural University, Zhengzhou, 450002 Henan China; 2 Collaborative Innovation Center of Henan Grain Crops, Zhengzhou, 450002 Henan China; 3 National Key Laboratory of Wheat and Maize Crop Science, Zhengzhou, 450002 Henan China; 4 Department of Entomology, Rutgers University, New Brunswick, 08901 New Jersey, USA

## Abstract

Burrowing nematodes (*Radopholus similis*) cause severe harm in many agronomic and horticultural crops and are very difficult to manage. Cathepsin S is one of the most important cysteine proteinases and plays key roles in nematodes and many other parasites. To evaluate the effect of *in planta* RNAi on the control of this nematode, a specific fragment from the protease gene, cathepsin S (*Rs*-*cps*), was cloned into the binary vector pFGC5941 in the forward and reverse orientations to construct recombinant plant RNAi vectors. Transgenic *Nicotiana benthamiana* plants expressing *Rs*-*cps* dsRNA were obtained and studied. The transcript abundance of *Rs*-*cps* dsRNA appeared to be diverse in the different transgenic lines. Moreover, the bioassay results revealed that *Rs-cps* transgenic *N. benthamiana* plants were resistant to *R*. *similis* and the transcription level of *Rs-cps* in *R*. *similis* was drastically decreased. In addition, the reproduction and hatching rate of *R*. *similis* isolated from the *Rs-cps* transgenic plants were also significantly reduced. Our results suggest that *Rs-cps* is essential for the reproduction and pathogenicity of *R*. *similis*. This is the first study to employ *in planta* RNAi approach to target the *Rs-cps* gene for the control of plant parasitic nematodes.

## Introduction

*Radopholus similis* is a migratory endoparasitic nematode that is known to be a destructive pest of bananas, peppers, coffee, citrus crops and many other agronomic and horticultural crops^1–4^. Both juvenile and adult nematodes can enter and leave root tissues and feed on the cytoplasm of cortex cells. Infection by *R*. *similis* deprives host plants of essential nutrients, whilst entry wounds make host roots more susceptible to other pathogens present in the soil. This destruction of crops leads to significant growth reduction and severe economic losses^5,6^. Although *R*. *similis* does great damage to agriculture, effective measures to control it are still lacking. The focus of control strategies for plant parasitic nematodes has depended on the application of expensive and environmentally unfriendly chemical nematicides^7,8^. Therefore, it is particularly important to explore effective approaches for controlling the nematode. One such strategy involves the application of plant-mediated RNA interference (*in planta* RNAi), which confers resistance to plants engineered to express specific dsRNA to target and silence specific genes involved in the reproduction, development, parasitism and pathogenesis of nematodes.

RNAi is an effective gene-silencing mechanism in eukaryotes, which was first discovered in *Caenorhabditis elegans*, and has provided a significant new tool to study gene function in many organisms including nematodes, plants, fungi, insects and mammals^8–16^. The dsRNA produced by transgenic plants against key pest genes has been regarded as a safeguard that endows transgenic pest-resistant plants with new innovations^17,18^. In recent years, *in planta* RNAi has emerged as an efficient tool to research gene functions and manage crop pathogens^17–24^. It also has been used to study gene functions in plant parasitic nematodes. The first successful demonstration of *in planta* RNAi was accomplished by targeting splicing factors and integrase genes of *Meloidogyne incognita*. Transgenic *Nicotiana benthamiana* plants constitutively expressing special dsRNA of these genes resulted in a reduction of root knots^25^. In another report, transgenic *Arabidopsis* expressing 16D10 dsRNA had a wide resistance against four major root-knot nematode species^19^. Some of the nematode genes were knocked down using *in planta* RNAi, causing reduction in the parasitic success of cyst and root-knot nematodes in different plants^26^. However, there are limited reports about the use of *in planta* RNAi in research against migratory plant parasitic nematodes^27–29^. Therefore, the further use of the *in planta* RNAi method to research the functions of parasitic or pathogenesis-related genes will enrich the understanding of the use of RNAi against migratory endoparasites and lay the foundation for the control of these nematodes.

Cysteine proteinases are essential for a wide range of physiological processes in all living organisms^30^. Cysteine proteinases play key roles in embryogenesis, development, invasion, parasitism and evasion of host immune responses, and most of them are the main digestive enzymes in the intestines of nematodes and many other animal parasites^16,31^. Therefore, these were identified as the primary targets for the control of parasites. In parasitic helminths, the papain superfamily of cysteine proteinases (i.e., cathepsins) has drawn the most attention^32^. According to the absence and presence of a distinctive set of amino acids within the polypeptide, there are more than 10 cathepsins within the cysteine proteinase family, including cathepsin B, C, L, S, F, K, and Z^33^. The most studied are cathepsin B and cathepsin L-like proteases, which have been studied in many parasitic nematodes in recent years. For the functional analysis of cathepsin genes in plant parasitic nematodes, special dsRNA delivery was accomplished by soaking the nematodes in a dsRNA solution (*in vitro* RNAi). As reported by Li *et al*., silencing of the cathepsin B gene (*Rs-cb-1*) using *in vitro* RNAi not only significantly inhibited the reproduction and development of *R*. *similis* but also greatly reduced its pathogenicity^29,34^. Targeting the cysteine proteinases of *Globodera pallida gpcp-*I and *Heterodera glycines hgcp-*I led to a decreased recovery of egg-laying females and altered sexual fate^11^. When *in vitro* RNAi was used to research the gene function of *Mi-cpl-1* in *M*. *incognita*, a reduction in gene transcript abundance was observed and the number of nematodes infecting plants was reduced by almost 60% at 21 days post-infection^35^. In our previous studies, we demonstrated that the cathepsin S gene of *R*. *similis* (*Rs*-*cps*) plays important roles in reproduction and pathogenesis using *in vitro* RNAi^16^. However, the functions of cathepsin S gene (cps) have rarely been researched, and only the cps genes of *H*. *glycines* and *H*. *avenae* have been cloned in other plant nematodes^30,31^.

Despite reports of *in vitro* RNAi studies, cps genes of plant parasitic nematodes have not yet been targeted using the *in planta* RNAi approach. At the same time, *Rs*-*cps* gene plays important roles in the reproduction and pathogenesis of *R*. *similis* ^16^. Therefore, we selected *Rs*-*cps* gene as a promising target for *in planta* RNAi research to control *R*. *similis*. In this study, the plant expression vector pFGC-Rs-cps2 was constructed, which can generate a hairpin RNAi construct. *Nicotiana benthamiana* transgenic lines producing *Rs*-*cps* dsRNA were generated from transformed callus tissues by *Agrobacterium-*mediated transformation. Putative transgenic *N. benthamiana* plants were detected by PCR, Southern blot and RT-PCR. In addition, single-copy *Rs-cps* transgenic plants were chosen for resistance studies. The feeding bioassay clearly showed that the resistance of *Rs-cps* transgenic *N. benthamiana* plants to *R*. *similis* was significantly improved, and the transcription level of *Rs-cps* in *R*. *similis* was drastically suppressed. The reproduction and hatching of *R*. *similis* isolated from *Rs-cps* transgenic *N. benthamiana* plants were also significantly inhibited. This is the first study to employ *in planta* RNAi targeting of the *Rs-cps* gene to control the plant parasitic nematode.

## Results

### Construction of plant RNAi expression vectors and production of transgenic plants

**Figure 1:**
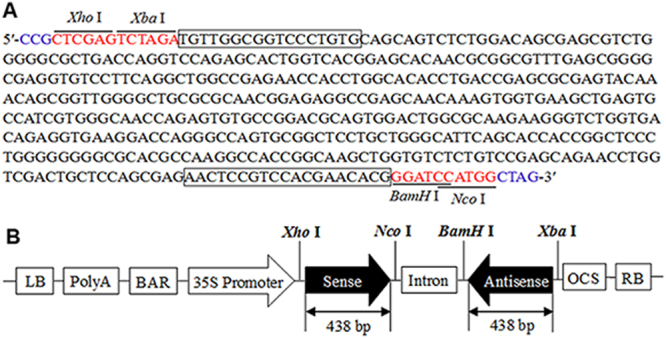
Target sequence of the *Rs-cps* gene for RNA interference and plant RNAi vector for *Nicotiana benthamiana* genetic transformation. (**A**) The sequence of *Rs-cps* was used for RNAi target sequences in this study. Blue font: protective bases; the specific primers are indicated in boxes. (**B**) Schematic representation of the plant RNAi vector expressing hairpin *Rs-cps* dsRNA in transgenic *N. benthamiana* plants.

A 438-bp partial cDNA fragment of *Rs-cps* was chosen as the target for RNAi (Fig. 1A). The constructed plant RNAi vector pFGC-Rs-cps2 contained a 438-bp sense and antisense *Rs-cps* cDNA fragment, a CHSA intron, an OCS terminator, and the cDNA fragments as inverted repeats under the control of CaMV35S promoter to produce the hairpin *Rs-cps* dsRNA (Fig. 1B). The constructed vectors were introduced into *Agrobacterium tumefaciens* strain EHA105 via the freeze-thaw method. The aseptic seedling was cut into small pieces of approximately 0.5 cm × 0.5 cm after four leaves grew, and the veins were removed. After pre-culture, the plant RNAi vectors were introduced into *N. benthamiana* explants by Agrobacterium-mediated transformation. The transgenic seedlings germinated from transformed *N. benthamiana* calli were selected for kanamycin resistance, and then, the seedlings were transferred into the rooting medium when they grew to approximately 2 cm. After acclimatization, the transgenic plantlets were transplanted into sterilized nutritive soil in a greenhouse for normal growth. We obtained a total of 26 independent kanamycin-resistant transgenic plants, including 15 *Rs-cps* transgenic plants and 11 egfp transgenic plants. These transgenic plants had wild-type *N. benthamiana* morphology and growth (result not shown).

### Molecular analysis of *Rs-cps* transgenic *N. benthamiana* plants

**Figure 2:**
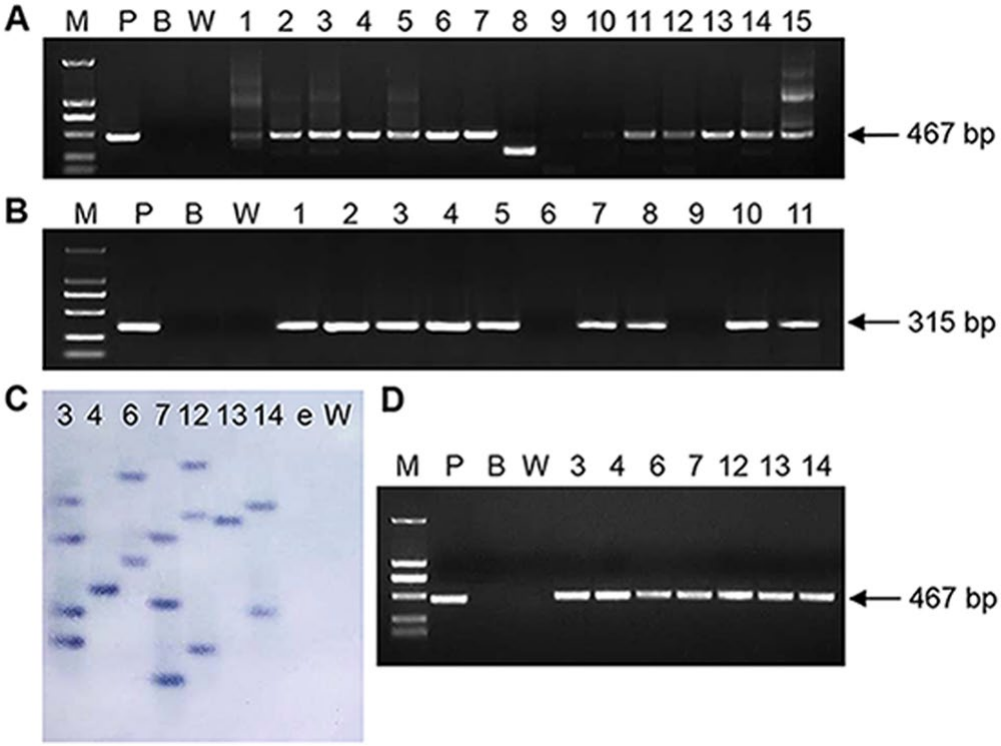
Molecular analysis of the putative transgenic *Nicotiana benthamiana* plants. (**A**) PCR analysis for putative *Rs-cps* transgenic plants using the primers RNAi-F/RNAi-R (lanes 1-15: different *Rs-cps* transgenic lines). (**B**) PCR analysis for putative e*gfp* transgenic plants using the primers eGFP-F/eGFP-R (lanes 1-11: different e*gfp* transgenic lines). (**C**) Southern blot analysis of *EcoR*I-digested genomic DNA from leaves of the T0 generation transgenic plants (lanes 3, 4, 6, 7, 12, 13 and 14: DNA from the 3, 4, 6, 7, 12, 13 and 14 *Rs-cps* transgenic lines; lanes e and W: DNA from the e*gfp* transgenic plant and wild**-**type *N. benthamiana* plant as the control). (**D**) Different transgenic plants were analysed by RT-PCR using the primers RNAi-F/RNAi-R (lanes 3, 4, 6, 7, 12, 13 and 14: RNA from the 3, 4, 6, 7, 12, 13 and 14 *Rs-cps* transgenic *N. benthamiana* plants). M, DL2000 DNA marker; P, positive plasmid control; B, blank control without template; W, wild-type *N. benthamiana* plant (negative control).

The independently generated T0 generation transgenic lines were analysed by PCR. A 467-bp DNA fragment of the target gene was amplified from most of the putative T0 generation *Rs-cps* transgenic plants, and a 315-bp DNA fragment was amplified from most of the putative *egfp* transgenic plants, while no specific band was amplified from the wild-type *N. benthamiana* plants (Fig. 2A,B). These results showed that the target DNA fragment was successfully inserted into the *N. benthamiana* genomic DNA. Although 13 of the 15 the kanamycin-resistant transgenic plants test positive for the RNAi construct in PCR, some detected plants showed nonspecific bands. Therefore, we only select part of transgenic plants (Nos. 3, 4, 6, 7, 12, 13 and 14) with specific bands for the Southern blot. The results showed that these *Rs-cps* transgenic plants had one to four insertion loci (Plants No. 4 and 13 had a single insertion locus, plants No. 6 and 14 had two insertion loci, plants No. 7 and 12 had three insertion loci, and plant No. 3 had four insertion loci), but no *Rs-cps* hybridization bands were observed with genomic DNA from the e*gfp* transgenic plants and wild-type *N. benthamiana* plants (Fig. 2C). RT-PCR was performed to detect the expression of *Rs-cps* dsRNA in positive transgenic *N. benthamiana* lines (Nos. 3, 4, 6, 7, 12, 13 and 14). A 467-bp fragment corresponding to the sequence of *Rs-cps* was amplified from these positive *Rs-cps* transgenic plants, while no specific amplification was observed from the wild-type *N. benthamiana* plants (Fig. 2D). These results indicated that the integrated *Rs-cps* dsRNA was successfully expressed in transgenic *N. benthamiana* plants. Genetic stability analysis indicated that the integrated *Rs-cps* could be inherited steadily in the genomic DNA of the T1 generation transgenic *N. benthamiana* plants (result not shown).

### Expression analysis of *Rs-cps* in T2 generation transgenic lines

**Figure 3:**
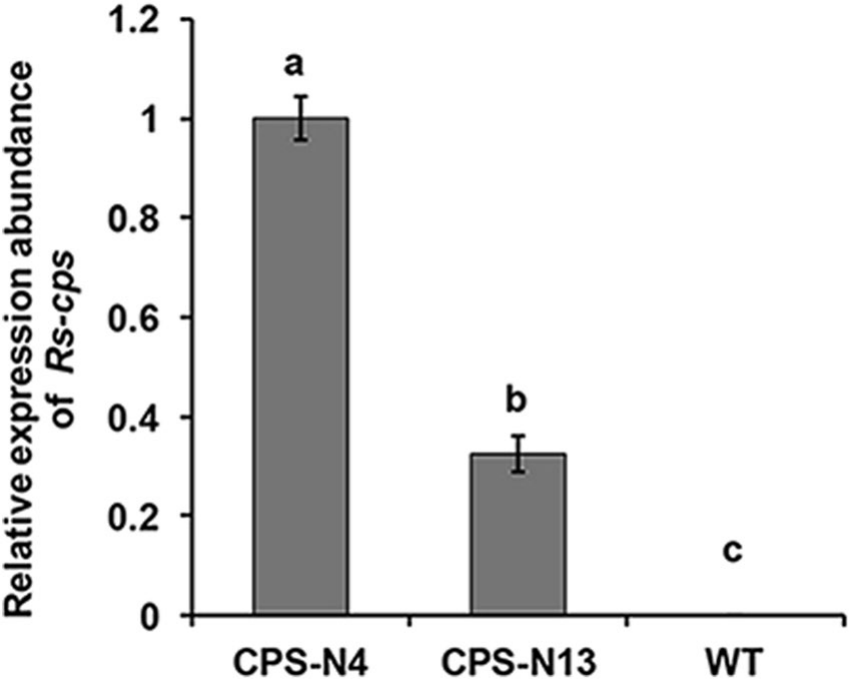
Expression analysis of *Rs-cps* in T2 generation transgenic plants. The mRNA abundance was determined by qPCR, and *Actin* was amplified as a reference gene. Bars indicate the standard errors of the mean data (n = 3), and different letters indicate significant differences (*p* < 0.05) between treatments. CPS-N4 and CPS-N13: No. 4 and No. 13 *Rs-cps* transgenic *Nicotiana benthamiana* plants; WT, wild-type *N. benthamiana* plants.

To detect the expression of the *Rs-cps* dsRNA transcript and its abundance, qPCR analysis of the selected single copy T2 generation transgenic lines (No. 4 and 13) was carried out. The result showed that *Rs-cps* dsRNA was expressed in two positive transgenic lines, while the transcript abundance of dsRNA varied between the two lines. The *Rs-cps* dsRNA expression level in line No. 4 was 3.07 times higher, which was significant (*p* < 0.05), than that in line No. 13 of the *Rs-cps* transgenic *N. benthamiana* plants (Fig. 3). Previous studies have shown that the effectiveness of RNAi is higher in single-copy lines than in other lines^36,37^. Therefore, the single-copy *Rs-cps* transgenic line (No. 4) with a relatively high expression level of dsRNA was selected for a nematodes feeding bioassay to analyse the reproduction and pathogenicity of *R*. *similis* and the efficiency of RNAi.

### T2 generation transgenic *N. benthamiana* plants expressing *Rs-cps* dsRNA show enhanced resistance to *R*. *similis*

**Figure 4:**
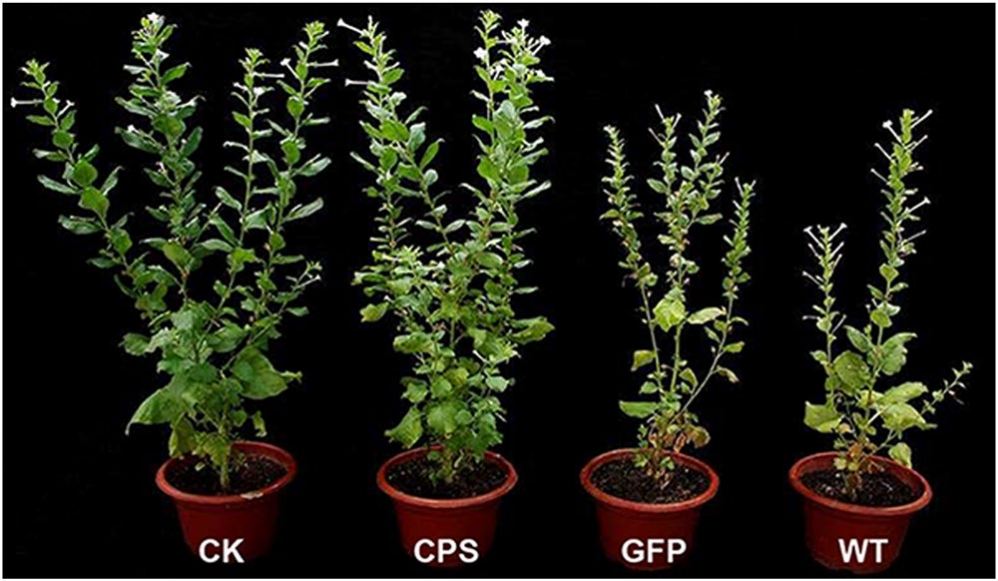
Infection symptoms of T2 generation transgenic plants after being inoculated with *R*. *similis* for 75 days. The selected *Nicotiana benthamiana* plantlets were approximately 20 cm in height, and each plantlet was inoculated with 2000 mixed stage nematodes and cultivated in a greenhouse. Seventy-five days after inoculation, *Rs-cps* transgenic plants with less damage exhibited higher resistance to *R*. *similis* than the control *N. benthamiana* plants. CK, uninoculated wild-type *N. benthamiana* plants; CPS, No. 4 *Rs-cps* transgenic plants; GFP, e*gfp* transgenic plants; WT, wild-type *N. benthamiana* plants.

**Figure 5:**
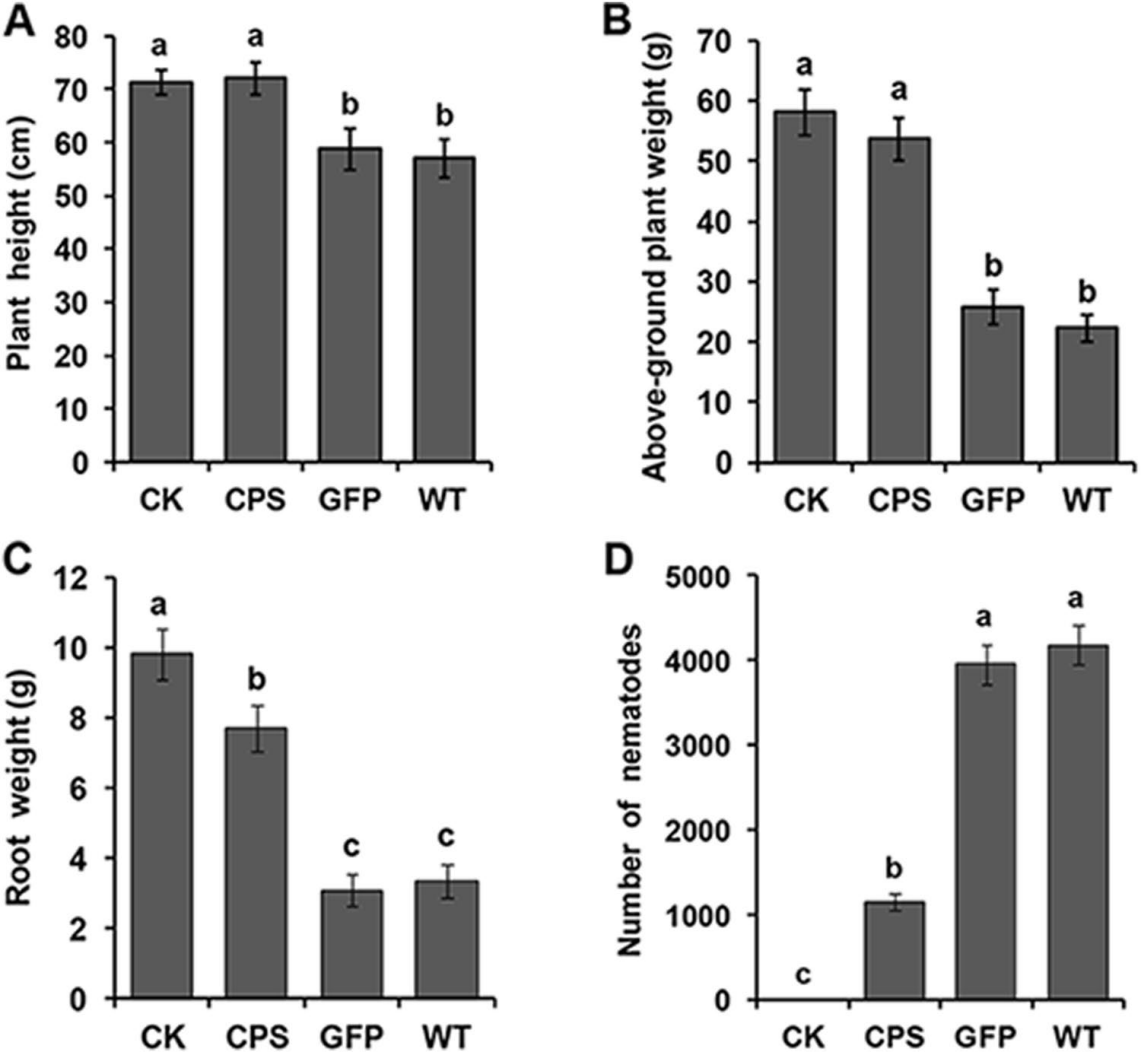
Resistance to *R*. *similis* in T2 transgenic *Nicotiana benthamiana* plants expressing *Rs-cps* dsRNA was significantly improved. Plant height (**A**), fresh above-ground plant weight (**B**), fresh root weight (**C**) and the number of nematodes in the rhizosphere (**D**) of the different *N. benthamiana* plants after being inoculated with 2000 mixed stage nematodes for 75 days. Bars indicate the standard errors of the mean data (n = 5), and different letters indicate significant differences (*p* < 0.05) between treatments. CK, uninoculated wild-type *N. benthamiana* plants; CPS, No. 4 *Rs-cps* transgenic plants; GFP, e*gfp* transgenic plants; WT, wild-type *N. benthamiana* plants.

To evaluate whether the resistance to *R*. *similis* was improved in T2 generation *Rs-cps* transgenic plants in comparison with e*gfp* transgenic plants and wild-type plants, nematodes were introduced to the roots of *N. benthamiana* plants. At 75 d, it was evident that the transgenic plants expressing *Rs-cps* dsRNA exhibited much higher resistance to *R*. *similis* than the control groups (e*gfp* transgenic plants and wild-type *N. benthamiana* plants). The control *N. benthamiana* plants were smaller and had fewer tillers than the *Rs-cps* transgenic plants. There were no obvious infection symptoms aboveground for the *Rs-cps* transgenic plants compared with the uninoculated wild-type *N. benthamiana* plants (Fig. 4). The pot experiment results showed that the plant height, fresh above-ground plant weight and fresh root weight of the *Rs-cps* transgenic plants (No. 4) were significantly higher than those in the control groups (*p* < 0.05). There was a significant difference in the fresh root weight between uninoculated wild-type and *Rs-cps* transgenic *N. benthamiana* plants (*p* < 0.05); however, no significant differences in the plant height and fresh above-ground plant weight were observed between them (*p* > 0.05). There was no significant difference in the three growth parameters between the control groups (*p* > 0.05) (Fig. 5A–C). Additionally, the number of nematodes in the rhizosphere of the *Rs-cps* transgenic plants was 1158, which was significantly lower than that of the e*gfp* transgenic plants (3954) and wild-type *N. benthamiana* plants (4182) (*p* < 0.05) (Fig. 5D). The pot inoculation trials clearly demonstrated resistance to *R*. *similis* in the T2 generation *Rs-cps* transgenic *N. benthamiana* plants was significantly improved.

### Transcription of *Rs-cps* in tested *R*. *similis* was dramatically suppressed by T2 generation transgenic *N. benthamiana* plant-derived *Rs-cps* dsRNA

**Figure 6:**
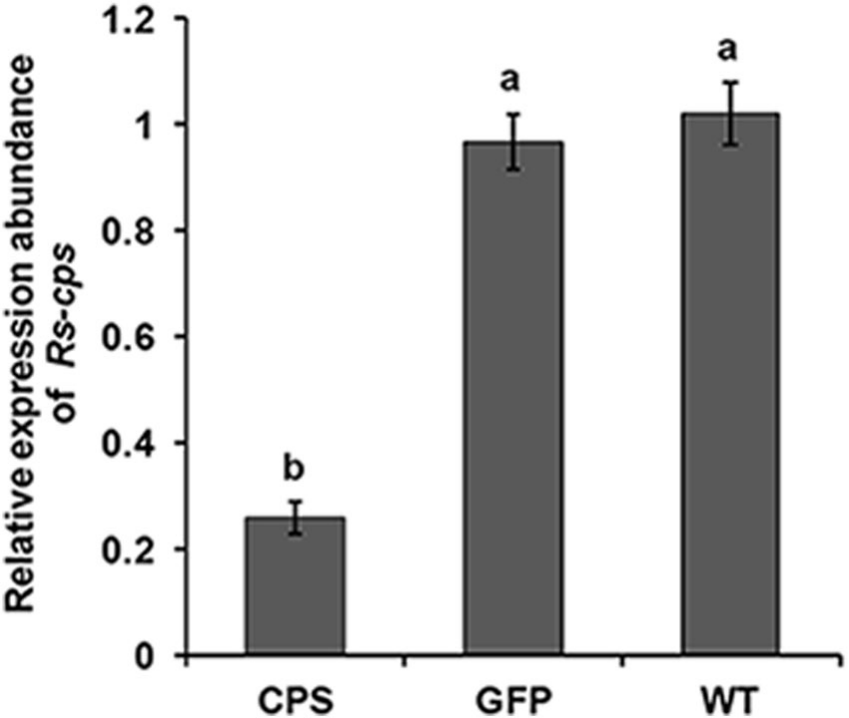
*Rs-cps* expression in *R*. *similis* was significantly suppressed by transgenic *Nicotiana benthamiana* plants-derived *Rs-cps* dsRNA. The mRNA abundance was determined by qPCR analysis, and *Actin* was amplified as a reference gene. Bars indicate the standard errors of the mean data (n = 3), and different letters indicate significant differences (*p* < 0.05) among groups. CPS, No. 4 *Rs-cps* transgenic plants; GFP, e*gfp* transgenic plants; WT, wild-type *N. benthamiana* plants.

We assume that the significant reduction in pathogenicity of *R*. *similis* resulted from suppression of its *Rs-cps* mRNA levels by feeding on transgenic plants expressing *Rs-cps* dsRNA. To confirm this hypothesis, the *Rs-cps* mRNA levels in *R*. *similis* isolated from the transgenic *N. benthamiana* roots were detected by qPCR. The results showed that (Fig. 6) the transcription level of *Rs-cps* in *R*. *similis* isolated from transgenic *N. benthamiana* plants expressing *Rs-cps* dsRNA was significantly lower (*p* < 0.05) than that from the control groups (e*gfp* transgenic plants and wild-type *N. benthamiana* plants) and was as much as 74.5% lower than that from wild-type *N. benthamiana* plants. There was no significant (*p* > 0.05) difference in the *Rs-cps* expression between the two control groups. Taken together, we conclude that the suppression of *Rs-cps* expression in *R*. *similis* by feeding on the roots of transgenic plants expressing *Rs-cps* dsRNA causes weaker pathogenic ability.

### Transgenic *N. benthamiana* plants expressing *Rs-cps* dsRNA inhibited the reproduction and hatching of *R*. *similis*

**Figure 7:**
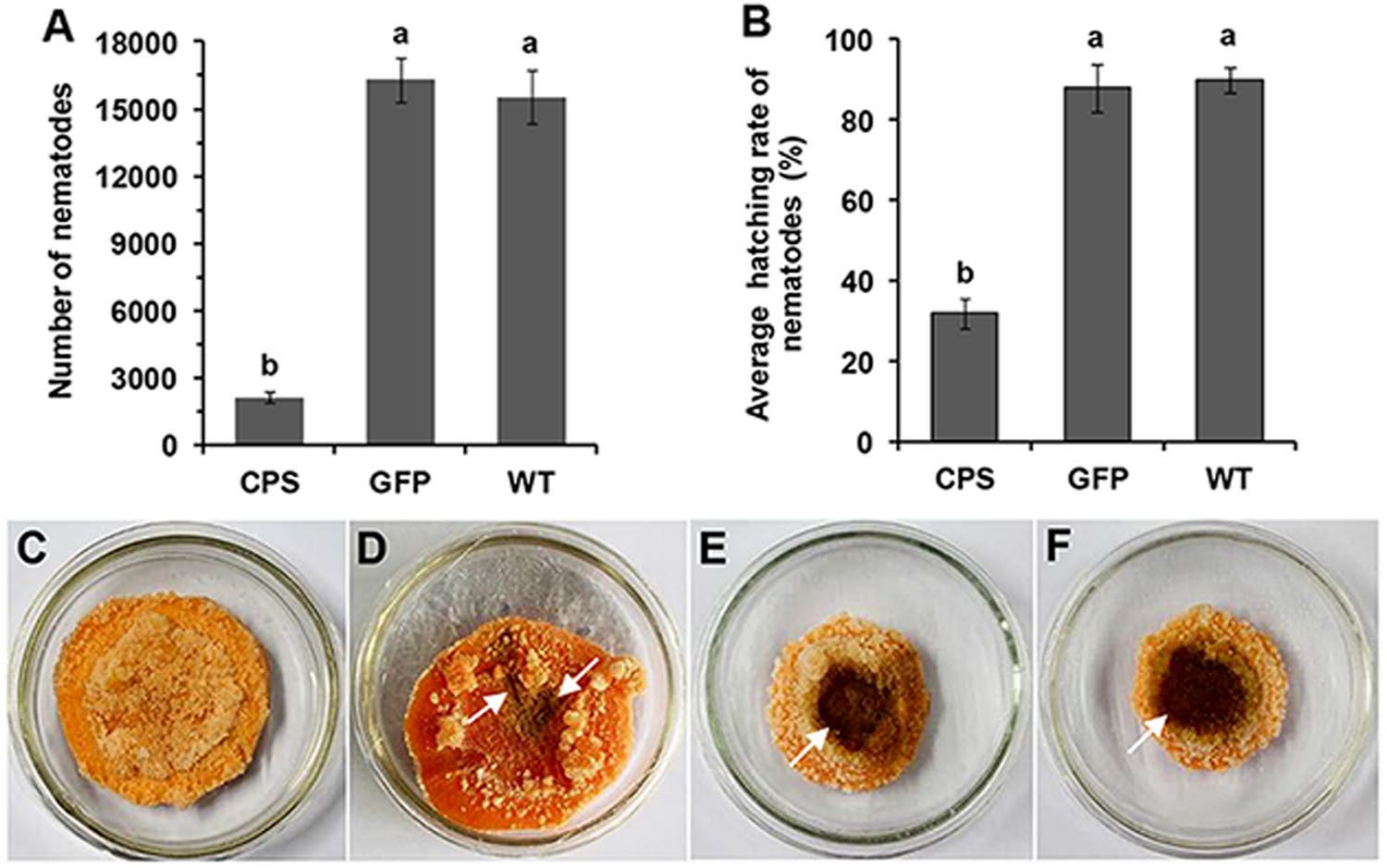
The reproduction and hatching of *R*. *similis* were decreased significantly by *Rs-cps* transgenic *Nicotiana benthamiana* plant-derived dsRNA. (**A**) The number of nematodes on carrot callus 50 d after the inoculation of 30 females isolated from No. 4 *Rs-cps* transgenic *N. benthamiana* roots. (**B**) The average hatching rate of eggs derived from No. 4 *Rs-cps* transgenic *N. benthamiana* plants. Bars indicate the standard errors of the mean data (n = 5), and different letters indicate significant differences among the different treatments (*p* < 0.05). Infection symptoms of carrot callus 50 d after being inoculated with 30 females isolated from No. 4 *Rs-cps* (**C**) and e*gfp* (**D**) transgenic *N. benthamiana* plants and wild-type (**E**) *N. benthamiana* plants. (**F**) Blank control without nematodes.

As described previously, the reproductive capability of *R*. *similis* treated with *Rs-cps* dsRNA (*in vitro* RNAi) was significantly decreased compared with the control groups^16^, and the number of nematodes in the rhizosphere of the *Rs-cps* transgenic plants was significantly lower than that of the control groups. To evaluate the effect of transgenic *N. benthamiana* plant-derived dsRNAs (*in planta* RNAi) on the reproduction and hatching of *R*. *similis*, the nematodes were inoculated onto carrot callus. After being cultured for 50 d, the nematodes isolated from the roots of *Rs-cps* transgenic *N. benthamiana* plants exhibited significantly lower reproduction than those from the roots of e*gfp* transgenic and wild-type *N. benthamiana* plants. The number of nematodes in the *Rs-cps* transgenic *N. benthamiana* groups (CPS) was 2136, which was significantly (*p* < 0.05) lower than those in the e*gfp* transgenic *N. benthamiana* groups (16296) and wild-type *N. benthamiana* groups (15536), but there was no significant (*p* > 0.05) difference observed between the latter two groups (Fig. 7A). In terms of the infection symptoms, the carrot callus presented brown hygrophanous lesions because of the reproduction of a large number of nematodes derived from the roots of e*gfp* transgenic and wild-type *N. benthamiana* plants. The carrot callus inoculated with nematodes derived from *Rs-cps* transgenic *N. benthamiana* roots only presented a slightly brown infection spot, and the brown hygrophanous lesion was not observed in this experiment (Fig. 7C–F). The hatching rate of eggs derived from *Rs-cps* transgenic *N. benthamiana* plants was 32%, which was significantly lower (*p* < 0.05) than that in the control groups (eggs derived from e*gfp* transgenic and wild-type *N. benthamiana* plants). The hatching rates of eggs derived from the e*gfp* transgenic and wild-type *N. benthamiana* plants were 88% and 90%, respectively, and there was no significant difference (*p* > 0.05) between these groups (Fig. 7B). All these results suggested that the *Rs-cps* transgenic plant-derived dsRNAs not only inhibited reproduction but also suppressed the hatching of *R*. *similis*.

## Discussion

Using RNAi in crop pathogens and pest control to suppress target genes using specific dsRNA has been well documented in recent years. Among the early studies, dsRNA was usually delivered by ingestion^20^, the injection method^38^ and feeding via bacteria expressing dsRNAs^39,40^. In spite of the huge costs, the injection method was still widely used for insect pests due to its high efficiency and accuracy. However, using a similar method for plant parasitic nematodes has been a major challenge. The introduction of special dsRNAs into plant parasitic nematodes could not be achieved consistently using microinjection^26^. The ingestion method (performed by soaking with synthesized dsRNA *in vitro*) and another approach to deliver special dsRNA into nematodes both showed potential for application in nematode control once special dsRNA was verified to down-regulate target gene expression. However, the efficiency of the latter two methods may not be as satisfactory for the instability of dsRNA when exposed to diverse environments. As a result, the above three methods are not applicable for the control of plant parasitic nematodes in farmland conditions. As an alternative, feeding the nematodes with transgenic plants expressing special dsRNA has been developed as an effective method to deliver dsRNA and control plant parasitic nematodes in agriculture. Transgenic plants possess relatively stable expression level of dsRNAs, providing nematodes with continual stress. Using *in planta* RNAi against plant parasitic nematodes was first described for root-knot nematodes^41^. It has also been used to study the control methods of many nematodes, including *M*. *incognita* ^19,26,42,43^, *M*. *javanica* ^44^, *H*. *glycines* ^45,46^, *R*. *similis* ^28,29^ and *Pratylenchus vulnus* ^27^. However, using an *in planta* RNAi method to target the *cps* gene for the control of plant parasitic nematodes has not yet been reported.

In this study, the *cps* gene from *R*. *similis* was selected as the special dsRNA construct, which was then transferred into *N. benthamiana* plants. The exact length of the dsRNA fragment needed to trigger RNAi in plants and other eukaryotes is still not entirely clear^47^. The targeting sequence for gene silencing in plants is approximately 300 to 700 bp in length^48^. In feeding experiments, most sequences range from 300 to 520 bp^49^. Previous research has shown that long dsRNA sequences may lead to greater RNAi effect compared to short dsRNAs^50^. Therefore, in the present study, a 438-bp partial cDNA sequence of *Rs-cps* was chosen as the target fragment for RNAi. As reported by Mao *et al*.^51^, the long dsRNA produced in plants could effectively suppress insect gene expression. Our study also showed that the transcription of *Rs-cps* in tested *R*. *similis* was dramatically suppressed by T2 generation transgenic *N. benthamiana* plant-derived *Rs-cps* dsRNA. Therefore, the result of the *Rs-cps* expression in *R*. *similis* suggests that the use of the 438-bp target fragment was successful in generating RNAi in this study.

Cysteine proteinases play key roles in nematodes and many other animal parasites^31^. Nematode cysteine proteinases mainly include cathepsin B-, L-, S-, K-, and Z-like cysteine proteinases, and cathepsin L and cathepsin B have been extensively studied in recent years^29,31,33–35^. Cathepsin S is one of the most important cysteine proteinases in nematodes and many other parasites. However, the functions of cathepsin S have not been researched using *in planta* RNAi in plant nematodes. This study was designed to investigate the functions of *Rs-cps* in *R*. *similis* and to find new targets for its control. Therefore, we used the similar method and strategy in this manuscript as described previously. The strategy of *in planta* RNAi against plant nematodes has been tested in several economically important crops^25,28,33,44–46^. All these works were using *in planta* RNAi to study gene functions of plant nematodes. However, only one economically important crop has been tested in each study. What’s more, the main purpose of this study is to investigate the functions of *Rs-cps* and to find new targets for its control. Therefore, we used a single model plants (*N. benthamiana*) for the RNAi research in this study. Previous studies have shown that the effectiveness of RNAi is higher in single-copy lines than in other lines^36,37^. Our results showed that No. 4 and No. 13 *Rs-cps* transgenic *N. benthamiana* plants had a single copy insertion. The expression level of *Rs-cps* dsRNA in line No. 4 was significant (*p* < 0.05) higher than that in line No. 13 of the *Rs-cps* transgenic plants. Therefore, we only selected the higher expression single-copy No. 4 *Rs-cps* transgenic line for the nematodes feeding bioassay.

*Rs*-*cps*, the target gene chosen in this study, plays important roles in the reproduction and pathogenesis of *R*. *similis*. In our previous studies, we verified the feasibility of RNAi for the *Rs-cps* gene in *R*. *similis* by soaking with *in vitro* synthesized dsRNA^16^. Therefore, the *Rs*-*cps* gene is expected to be a promising target for controlling *R*. *similis*. In this study, *N. benthamiana* plants were transformed to express the special *Rs*-*cps* dsRNA and challenged with nematodes to research the *Rs*-*cps* function in *R*. *similis* using *in planta* RNAi. Previous studies have shown that delivering dsRNA to plant parasitic nematodes by feeding them with transgenic plants results in the reduction of the target mRNA expression by approximately 65% to 80%^28,29,44^. In this study, the transcription level of *Rs-cps* in *R*. *similis* fed with *Rs-cps* transgenic *N. benthamiana* plants was significantly reduced by 74.5% compared with the control group (*p* < 0.05). However, this dsRNA-mediated RNAi did not completely eliminate expression of the gene products. That is, the target genes can be knocked down, rather than knocked out, through plant-mediated RNAi. This was consistent with the results seen using *in vitro* RNAi-mediated gene silencing^11,14–16^. There are several possible reasons for this phenomenon. The first reason for the phenomenon may be due to the lower accumulation of dsRNA in the transgenic plants. Dicer-like enzymes in plants could have decreased the expression levels of the generated dsRNA in these transgenic lines^47^. The second reason may be that there are energy metabolic pathways that compensate for the RNAi targeting genes.

In this study, we confirmed that the resistance of T2 generation *Rs-cps* transgenic *N. benthamiana* plants to *R*. *similis* was significantly improved. The result was consistent with the roles of *Rs-cps* in *R*. *similis*, which was verified by *in vitro* RNAi^16^. *In planta* RNAi has been used to research gene functions in cyst nematodes, root-knot nematodes, *R*. *similis* and other plant parasitic nematodes. As reported by Klink *et al*.^46^, *in planta* RNAi targeting of four genes of *H*. *glycines* caused 81–93% fewer females to develop in transgenic soybean roots. In another report, transgenic *Arabidopsis* expressing 16D10 dsRNA had a wide resistance against four major root-knot nematode species^19^. Steeves *et al*. demonstrated that MSP transgenic soybean plants significantly reduced the reproductive potential of *H*. *glycines* ^45^. All these studies were done using *in planta* RNAi to research gene functions and demonstrate the efficacy of an RNAi-based strategy for controlling sedentary plant parasitic nematodes. There is limited information available regarding *in planta* RNAi against migratory endoparasites compared to sedentary endoparasites, even though Li *et al*. reported that the T2 generation transgenic plants expressing special dsRNA show enhanced resistance to *R*. *similis* ^28,29^. In this study, we first confirmed the feasibility of *in planta* RNAi in controlling *R*. *similis* by targeting the *Rs-cps* gene. Steeves *et al*.^45^ first confirmed the inheritability of gene silencing induced by *in planta* RNAi in the sedentary plant nematode *H*. *glycines*. In another report, burrowing nematodes infecting transgenic *N. benthamiana* plants expressing specific dsRNA targeting the *Rs-cb-1* gene showed a significant reduction in pathogenicity. Interestingly, the progeny nematodes (F1 generation) that hatched from the eggs also showed a significant reduction in pathogenicity and reproduction when allowed to infect the wild-type *N. benthamiana* plants. Our study showed that the hatching rate of eggs derived from *Rs-cps* transgenic plants was 32%, which significantly lower than that in control groups. The result was consistent with the inheritability of gene silencing induced by *in planta* RNAi described above. In conclusion, this study demonstrated that specific dsRNA targeting *Rs-cps* was effective at suppressing the reproductive capability and hatching of *R*. *similis*, and the resistance to *R*. *similis* was significantly improved in *Rs-cps* transgenic *N. benthamiana* plants. To the best of our knowledge, this is the first study to report the use of an *in planta* RNAi approach targeting the *Rs-cps* gene to control *R*. *similis*. The current results will enrich the understanding of the use of RNAi against migratory plant parasitic nematodes and provide a scientific basis for the control of other nematodes and pests.

## Methods

### Plant materials and growth conditions

*Nicotiana benthamiana* was used as the background line for transformation in this study. For germination, seeds were soaked in sterile water for 1 h at 26 °C, treated with 75% (v/v) absolute ethanol for 1 min and with 0.75% NaOCl for 30 min, washed several times with sterile water, and then sowed on MS medium (pH 5.8) solidified with 0.3% phytagel^52,53^. The aseptic seeds germinated, and the seedlings were cultured in a growth chamber at 26 ± 1 °C under a photoperiod of 16 h-light /8 h-dark^22^.

### Culturing of nematodes

Carrot discs were prepared as previously described^54^. The banana burrowing nematode was collected from the roots of *Anthurium andraeanum* and cultured on carrot discs at 25 °C in a dark incubator^55^. Approximately 50 d after inoculation, the cultured nematodes were extracted from the carrot discs according to the method of Zhang *et al*.^14^.

### Cloning and sequencing of the *Rs-cps* gene

Total RNA was isolated from mixed-stage nematodes using TRIzol reagent (Invitrogen, USA) and digested with DNase (RQ1, Promega) to remove the contaminating DNA molecules. The quality of the extracted RNA was assessed using a spectrophotometer (Nanodrop ND-2000C, Thermo Scientific). The full-length cDNA sequence of *Rs*-*cps* was amplified using the specific primers *Rs-cps*-S1 and *Rs-cps*-A1 (Table 1), and the purified PCR product was cloned and confirmed by sequencing as previously described^16^. The recombinant pMD20-*Rs-cps* plasmid was extracted for later use.

**Table 1:** Primers used in this study.

Primer name	Sequences (5′ to 3′)	Primer use
Rs-cps-S1	AGTGCCCCTCCGAAATGT	cDNA amplification
Rs-cps-A1	TGTCCGTTCTTCCGTTCA	
RNAi-F	CCGCTCGAGTCTAGATGTTGGCGGTCCCTGTG	Vector construction
RNAi-R	CTAGCCATGGATCCCGTGTTCGTGGACGGAGTT	
eGFP-F	CCGCTCGAGTCTAGATGCTTCAGCCGCTACCC	Vector construction
eGFP-R	CATGCCATGGATCCAGTTCACCTTGATGCCGTTC	
Southern-F	TGTTGGCGGTCCCTGTG	Southern blot
Southern-R	CGTGTTCGTGGACGGAGTT	
qPCR-F	GGTCACGGAGCACAACGCG	qPCR
qPCR-R	GGCACTCAGCTTCACCACTTT	
Nb-actin-F	CTGAGAGATTCCGCTGC	qPCR
Nb-actin-R	GAGGACAATGTTTCCGTAC	
Actin-F	GAAAGAGGGCCGGAAGAG	qPCR
Actin-R	AGATCGTCCGCGACATAAAG	

### Plant expression vector construction and *N. benthamiana* transformation

For the *Rs-cps* plant RNAi expression vector, the partial coding region of *Rs-cps* was amplified from the pMD20-*Rs-cps* plasmid containing the full-length *Rs-cps* cDNA using the specific primers RNAi-F and RNAi-R (Table 1)^16^. *Xho*I and *Xba*I restriction enzyme sites were added to the 5′ end of the forward primer RNAi-F. At the same time, *Nco*I and *BamH*I restriction enzyme sites were added to the 3′ end of the reverse primer RNAi-R. The digested *Rs-cps* fragments were inserted into the *Xba*I/*BamH*I and X*ho*I/*Nco*I enzyme sites of the binary vector pFGC5941 at inverted repeat sequences to form a plant RNAi expression vector, pFGC-Rs-cps2, which can generate a hairpin RNAi construct. A similar non-endogenous control vector, pFGC-egfp2, was constructed using the primers eGFP-F/eGFP-R (Table 1)^29^. Then, the constructed pFGC-Rs-cps2 and pFGC-egfp2 were introduced into the competent cells of *Agrobacterium tumefaciens* (EHA105) by the freeze-thaw method^56^. The co-cultivation of a *N. benthamiana* leaf disc was followed using previously described methods^57,58^. The plant transformation was performed as described previously^59^, and the transformants were selected for kanamycin resistance on MS medium. After one month of tissue culture, the well-rooted *N. benthamiana* plants were transferred into sterilized nutritive soil for normal growth and reproduction under the greenhouse conditions described above.

### Molecular analysis of putative transformed *N. benthamiana* plants

Putative transgenic *N. benthamiana* plant lines were identified by PCR, Southern blot hybridization and RT-PCR. Genomic DNA (gDNA) was extracted from the leaves of T0 generation transgenic and wild-type *N. benthamiana* plants using the cetyltrimethyl ammonium bromide (CTAB) method^60^. *Rs-cps* transgenic *N. benthamiana* plants were confirmed by PCR with the specific primers RNAi-F and RNAi-R (Table 1). The e*gfp* transgenic plants were similarly checked using the specific primers eGFP-F/eGFP-R as described above (Table 1). To confirm integration of the transformed plants, Southern blot hybridization was performed using the DIG High Prime DNA Labeling and Detection Starter Kit I (Roche). The extracted gDNA (15 μg) of each PCR-positive *N. benthamiana* line was digested with *EcoR*I (Thermo Scientific) at 37 °C and then transferred to a Hybond N+ membrane (Amersham)^29^. The DIG-labelled probe was prepared using the specific primers Southern-F/Southern-R (Table 1)^16^. Hybridisation and detection were performed as previously described^16,29^. Equal amounts of gDNA from the T0 generation e*gfp* transgenic and wild-type *N. benthamiana* plants were used as the controls. For the RT-PCR, total RNAs of PCR and Southern-positive *Rs-cps* transgenic *N. benthamiana* leaves were extracted and reverse transcribed using the PrimeScript^TM^ 1st Strand cDNA Synthesis Kit (Takara). The PCR reactions were carried out using the specific primers RNAi-F/RNAi-R (Table 1) as described above. The positive transgenic *N. benthamiana* plants were cultured in the greenhouse to obtain seeds for further research.

### Detection the transcription level of *Rs-cps* in transgenic *N. benthamiana* leaves by qPCR

qPCR was performed to detect the production of *Rs-cps* dsRNA in different single copy T2 generation transgenic and wild-type *N. benthamiana* plants. Total RNA of these fresh *N. benthamiana* leaves was extracted and reverse transcribed as described above. The specific primers qPCR-F and qPCR-R (Table 1) were designed to detect *Rs*-*cps* expression levels. The actin gene of *N. benthamiana* (GenBank accession No: AY179605) was amplified as a reference using the primers Nb-actin-F and Nb-actin-R (Table 1). qPCR tests were performed with a Mastercycler EP Realplex qPCR machine (Eppendorf) in a reaction volume of 20 μl using iTaq Universal SYBR Green Supermix (Bio-Rad). Data were collected at the end of each extension step. The initial data analysis was carried out using the manager software, which calculated Ct values, and the relative levels of *Rs-cps* were extrapolated from standard curves. Melt curves were obtained routinely, which allowed the possibility of both contamination and primer dimers to be discounted. All expression experiments were performed in triplicate with three biological replicates^29^.

### Resistance studies of T2 generation transgenic *N. benthamiana* plants expressing *Rs-cps* dsRNA against *R*. *similis*

To investigate the resistance of the transgenic plants, the single-copy *Rs-cps* transgenic line was chosen for the feeding bioassay. Wild-type *N. benthamiana* and e*gfp* dsRNA-expressing *N. benthamiana* lines were used as controls. A total of 2,000 mixed-stage nematodes were inoculated to the roots of each of the selected *N. benthamiana* plantlets, which had been grown under the same conditions (approximately 20 cm in height). Plants were harvested 70 d post-inoculation, and roots were washed free of soil. The infection symptoms, plant height, fresh above-ground plant weight and fresh root weight of these different inoculated *N. benthamiana* plants were measured and recorded. In addition, observations were taken on the number of nematodes in the rhizosphere for each *N. benthamiana* plant under a microscope as described previously^14,61^. There were 5 replicates for each experiment, and each experiment was conducted twice. Representative data from one round of the experiments were presented.

### Expression analysis of *Rs-cps* in *R*. *similis* extracted from T2 generation transgenic *N. benthamiana* plants

Approximately 100 mixed-stage nematodes feeding on the roots of the wild-type and transgenic *N. benthamiana* plants were isolated. Extracted nematodes were washed with DEPC water, frozen immediately in liquid N2 and stored at −80 °C. Total RNA was extracted using the RNeasy Micro Kit (Qiagen) and reverse transcribed as described above. Transcript accumulation of *Rs-cps* in *R*. *similis* was analysed by qPCR using the specific primers qPCR-F/qPCR-R and Actin-F/Actin-R (Table 1) as previously described^28,29^. All the results were obtained from three independent biological replicates. The relative gene expression data were analysed as described above.

### Analysis of reproduction and hatching of nematodes extracted from transgenic *N. benthamiana* plants

The female nematodes isolated from the roots of T2 generation *Rs-cps* transgenic *N. benthamiana* plants were sterilized with streptomycin sulfate (3 g/L) for 6 h and then washed with sterile water. After that, the sterilized nematodes were used for the following experiments: (I) A total of 30 females were inoculated onto carrot callus, and the reproduction was evaluated after 50 d at 25 °C. (II) A total of 100 females were cultured on carrot callus for 25 d to obtain nematode eggs. The collected eggs were observed and collected by a dropper under a Ti inverted microscope (Nikon), then placed in Petri dishes (3 cm in diameter, 10/dish) with sterile water and cultivated at 25 °C. After being cultured for 7 d, eggs were observed every 12 h till hatching, and the hatching rates (hatching rate = number of hatched eggs/total number of eggs) were calculated. Nematodes isolated from the roots of e*gfp* transgenic and wild-type *N. benthamiana* plants were used as the controls. There were 5 replicates for each experiment, and each experiment was conducted twice. Representative data from one round of the experiments were presented.

### Statistical analyses

All statistical analyses were conducted using SAS 9.2 (SAS Institute, Cary, NC, USA) in this study. The data collected from the experiments were subjected to one-way analysis of variance (ANOVA) and tested for differences among treatments at the 5% level using Duncan’s Multiple Range Test (DMRT).
